# Long-term functional outcomes and vision-related quality of life after vitrectomy for epiretinal membrane: a prospective cohort study

**DOI:** 10.1038/s41598-022-06482-3

**Published:** 2022-02-15

**Authors:** Raoul Kanav Khanna, Marta Dorvault, Jeremy Pasco, Ann-Rose Cook, Tiphanie Pichard, Marie-Thérèse Marotte, Pierre-Jean Pisella, Sophie Arsene

**Affiliations:** 1 Department of Ophthalmology, University Hospital of Tours, Tours, France; 2 INSERM 1253 iBrain « Neurogénomique & Physiopathologie neuronale », University Hospital of Tours, Tours, France; 3 Clinical Data Center, SIMEES, University Hospital of Tours, Tours, France

**Keywords:** Retinal diseases, Vision disorders, Macular degeneration

## Abstract

To investigate the long-term effect of unilateral idiopathic epiretinal membrane (uiERM) removal on monocular and binocular visual function, and on vision-related quality of life (VR-QoL). Prospective, single-center study. The following data were collected before and after surgery: distance monocular and binocular best-corrected visual acuity (BCVA), horizontal and vertical metamorphopsia, horizontal and vertical aniseikonia, stereoacuity and National Eye Institute Visual Function Questionnaire-25 item (NEI VFQ-25). Forty-two patients (mean age: 72.7 ± 7.4 years; 24 men) were included. At 6 months postoperatively, distance monocular BCVA (*p* < 0.001), horizontal metamorphopsia (*p* = 0.001) and the composite score of NEI VFQ-25 (*p* < 0.001) significantly improved, in comparison to baseline. At 2 years postoperatively, distance monocular (*p* < 0.001) and binocular (*p* = 0.01) BCVA, horizontal (*p* < 0.001) and vertical (*p* = 0.02) metamorphopsia, vertical aniseikonia (*p* = 0.01), stereoacuity (*p* < 0.001) and 3 subscales scores of the NEI VFQ-25 (*p* < 0.05) (“general vision”, “mental health”, “driving”) significantly improved in comparison to baseline. Removal of uiERM improves VR-QoL and achieves good visual outcomes on both monocular and binocular visual parameters over long-term. Visual symptoms induced by macular contraction have different improvement kinetics after surgery. Stereopsis, the highest level of binocular vision, can be improved in some cases.

## Introduction

Epiretinal membrane (ERM), first described by Jaffe in 1967 is a fibrocellular, avascular proliferation, with a contractile macular or paramacular epicenter, located anterior to the retinal surface. ERM may cause monocular functional symptoms in the affected eye (decrease in best corrected visual acuity (BCVA), positive central scotoma, metamorphopsia,) and lead to impaired binocular vision (aniseikonia, decreased stereoacuity)^1^. Multiple mechanisms have been suggested for the pathogenesis of these visual impairments: macular folds and distortions, macular displacement, vascular hyperpermeability causing macular edema, opacification of the visual axis by the ERM itself.

Severity in preoperative metamorphopsia and preoperative binocular distance BCVA were found to correlate with preoperative vision-related quality of life (VR-QoL)^2^. Yet, only one study performed multiple regressions and found a significant association between the severity in horizontal metamorphopsia and horizontal aniseikonia and decreased VR-QoL prior to surgery^3^. While BCVA and metamorphopsia have been shown to be significantly improved by ERM removal^2,4–14^, findings are inconsistent regarding binocular visual function parameters such as aniseikonia and stereoacuity. To our knowledge, there are no studies evaluating VR-QoL, monocular and binocular visual parameters after ERM removal with a postoperative follow-up period above 1 year, as the follow-up period in the current literature ranges from 3 (Okamoto et al.^2,6^) to 12 months (Nakashizuka et al. 2019^12^, Matsuoka et al. 2012^7^).

The main purpose of our study was to evaluate the outcomes after unilateral idiopathic ERM (uiERM) surgery on monocular and binocular visual function as well as on VR-QoL with a 2 years follow-up.

## Results

Data from 42 patients were analyzed (24 men; mean age: 72.7 ± 7.4 years). Of the 42 eyes included in the study, 35 (83%) eyes were phakic and 7 (17%) were pseudophakic prior to ERM surgery. Twenty-two phakic patients received combined cataract and ERM surgery during the same intervention. Eleven patients received a cataract operation on the eye affected by ERM during their postoperative follow-up (mean: 14.1 ± 9.5 months following ERM surgery), 7 patients between ERM surgery and 6 months postoperatively and 4 patients between 6 months and 2 years postoperatively. Two patients remained phakic in the eye operated on for ERM at the end of follow-up. The mean duration of symptoms before surgery was 13.6 ± 8.6 months. No intraoperative complications were reported. Four patients presented an Irvine-Gass syndrome successfully managed with appropriate medical treatment and did not present any macular edema at the time of data collection. The collection of postoperative data was carried out in average at 6.3 ± 2.5 months and 28.4 ± 3.6 months following surgery. At 2 postoperative years, 5 (12%) patients presented an idiopathic ERM in the contralateral eye, diagnosed by SD-OCT, without anatomical changes in macular morphology or retinal thicknesses. No patients presented a recurrence of ERM during follow-up on SD-OCT.

### Clinical and paraclinical parameters

**Figure 1:**
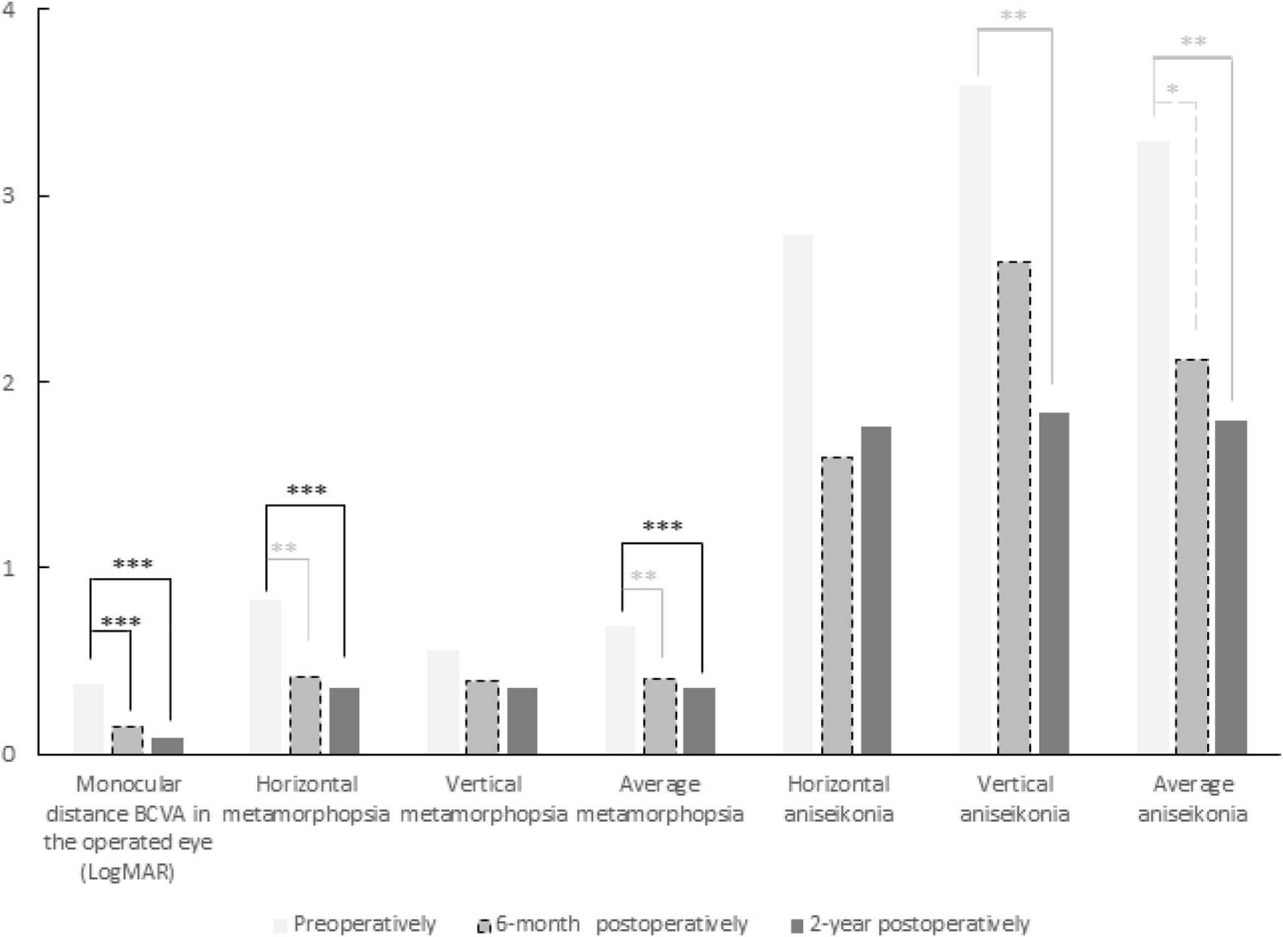
Evolution of the mean values of monocular distance best-corrected visual acuity (BCVA in LogMAR), metamorphopsia and aniseikonia preoperatively, 6-month postoperatively and 2-year postoperatively. The vertical axis indicates BCVA in LogMAR and values of metamorphopsia and aniseikonia using the M-CHARTS and NAT respectively; *p value < 0.05; **p value < 0.01; ***p value < 0.001.

The clinical and paraclinical parameters of the patients at the different times of follow-up are presented in Table 1. The evolution of monocular distance BCVA, metamorphopsia scores and aniseikonia scores are illustrated in Fig. 1.

**Table 1:** Clinical and paraclinical parameters prior to surgery, at 6 months and 2 years postoperatively.

	Preop	Postop 6 months		Postop 2 years	
			*p* value^†^		*p* value^‡^
Monocular distance BCVA in the operated eye (LogMAR), median (range)	0.4 (0.1 to 1.2)	0.1 (− 0.1 to 0.8)	**<** **0.001a**	0.1 (− 0.1 to 0.7)	**<** **0.001a**
Monocular distance BCVA in the healthy eye (LogMAR), median (range)	0 (− 0.3 to 0.2)	0 (− 0.3 to 0.2)	0.93a	0 (− 0.1 to 0.2)	0.49a
Binocular distance BCVA (LogMAR), median (range)	0 (− 0.2 to 0.4)	0 (− 0.3 to 0.2)	0.14a	0 (− 0.3 to 0.1)	**0.01a**
**Metamorphopsia score (M-CHARTS)**					
Horizontal, median (range)	0.7 (0 to 2)	0 (0 to 2)	**0.001a**	0 (0 to 2)	**<** **0.001a**
Vertical, median (range)	0.5 (0 to 2)	0.4 (0 to 2)	0.08a	0.3 (0 to 2)	**0.02a**
Average, median (range)	0.6 (0 to 2)	0.4 (0 to 1.6)	**0.001a**	0.2 (0 to 2)	**<** **0.001a**
**Aniseikonia (NAT)**					
Horizontal, median (range)	1 (0 to 16)	0 (0 to 10)	0.06a	1 (0 to 15)	0.12a
Vertical, median (range)	3 (0 to 24)	1 (0 to 18)	0.32a	1 (0 to 11)	**0.01a**
Average, median (range)	3 (0 to 13)	1 (0 to 14)	**0.03a**	1 (0 to 13)	**0.01a**
Macropsia, n (%)	22 (52%)	24 (57%)		29 (69%)	
Micropsia, n (%)	11 (26%)	3 (7%)		4 (9%)	
Absence of aniseikonia, n (%)	9 (22%)	15 (36%)		9 (22%)	
**Stereoacuity (arc-seconds), median (range)**	> 480 (240 to > 480)	> 480 (60 to > 480)	0.07a	480 (30 to > 480)	**<** **0.001a**
> 480 (none)	36 (86%)	28 (67%)		19 (45%)	
> 120″ et ≤ 480 (poor quality)	6 (14%)	6 (14%)		13 (31%)	
≥ 15″ et ≤ 120 (good quality)	0	7 (17%)*		10 (24%)	
**Central retinal thickness (µm), median (range)**	477 (335 to 644)	362 (269 to 472)	**<** **0.001a**	343 (272 to 406)	**<** **0.001a**
**Normal ellipsoid zone integrity**	25 (59.5%)	35 (83%)	**<** **0.001b**	37 (88%)	**<** **0.001b**

*BCVA* best-corrected visual acuity, *NAT* New Aniseikonia Test, *TNO* The Netherlands Organization for applied scientific research.

^†^*p* value between Preop and 6 months Postop; ^‡^*p* value between Preop and 2 years Postop; **In bold**: statistically significant difference (*p* < 0.05); a Wilcoxon signed-rank test; b McNemar test; *1 TNO data missing at 6 months postoperatively.

Preoperatively, the metamorphopsia score was significantly higher in the horizontal direction than in the vertical direction (*p* = 0.014). Contrariwise, the aniseikonia score was greater in the vertical direction than in the horizontal direction, without being significantly different (*p* = 0.47).

There was no significant difference in monocular distance BCVA in the healthy eye according to the different times of data collection: between the preoperative period and 6 months postoperatively (*p* = 0.93) and between the preoperative period and 2 years postoperatively (*p* = 0.49).

### Vision-related quality of life scores

**Figure 2:**
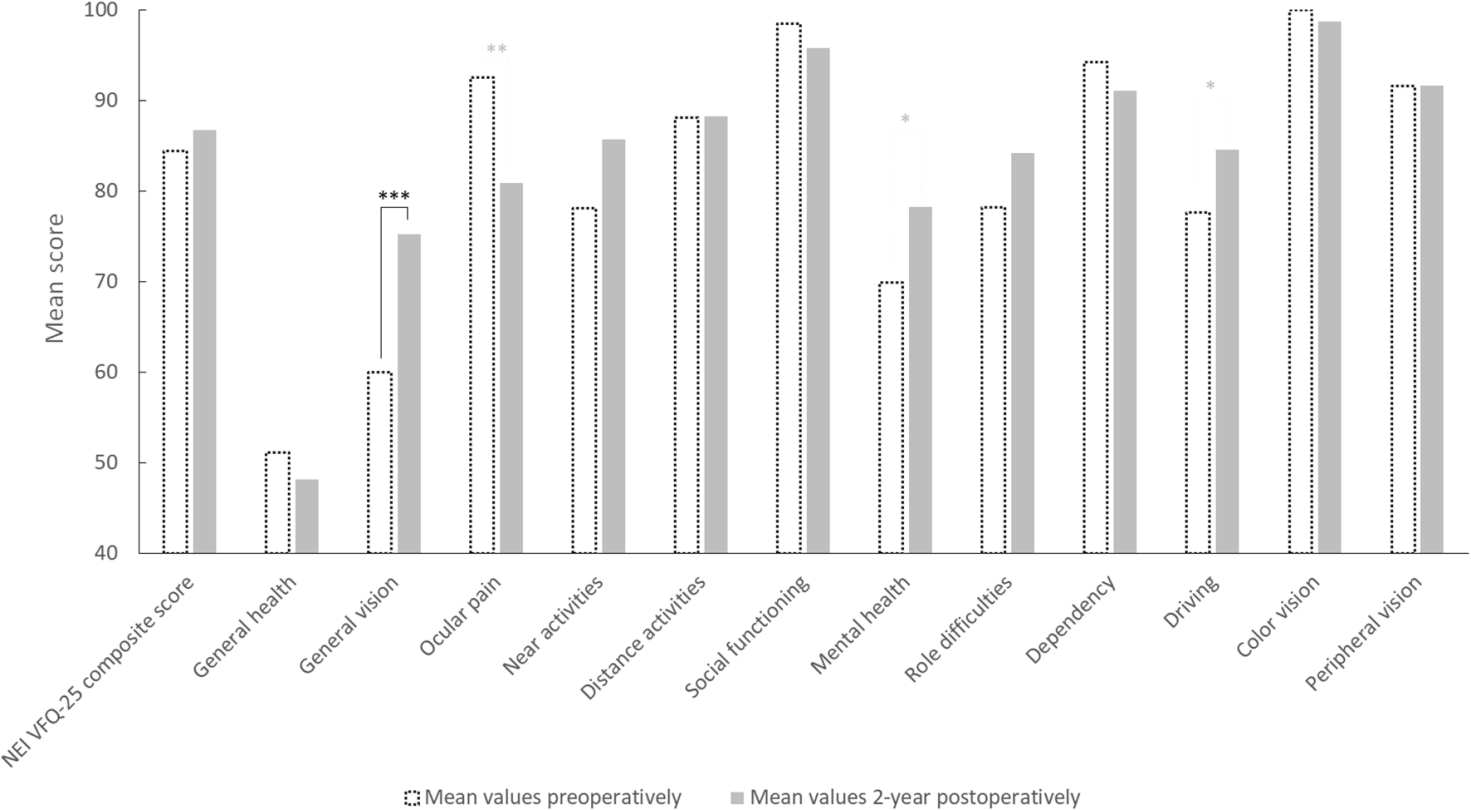
Evolution of the mean values of each sub-category and the composite score of the NEI VFQ-25 preoperatively and 2-year postoperatively. *p value < 0.05; **p value < 0.01; ***p value < 0.001.

VR-QoL scores prior surgery, at 6 months and 2 years postoperatively are summarized in Table 2. The evolution of the NEI VFQ-25 between preoperative assessment and 2 years postoperatively is illustrated in Fig. 2.

**Table 2:** NEI VFQ-25 composite score and scores of the 12 sub-categories, prior to surgery, at 6 months and 2 years postoperatively.

NEI VFQ-25 scores	Preop	6 months Postop		2 years Postop	
	Median (range)	Median (range)	*p* value^†^	Median (range)	*p* value^‡^
General health	50 (0 to 100)	50 (25 to 100)	**0.034**	50 (0 to 100)	0.202
General vision	60 (20 to 80)	80 (10 to 100)	**<** **0.001**	80 (60 to 100)	**<** **0.001**
Ocular pain	100 (38 to 100)	100 (100 to 100)	**0.005**	88 (50 to 100)	**0.002**
Near activities	83 (8 to 100)	100 (83 to 100)	**<** **0.001**	92 (33 to 100)	0.145
Distance activities	92 (50 to 100)	100 (92 to 100)	**<** **0.001**	92 (63 to 100)	0.417
Social functioning	100 (75 to 100)	100 (100 to 100)	0.068	100 (38 to 100)	0.161
Mental health	75 (19 to 100)	100 (75 to 100)	**<** **0.001**	81 (25 to 100)	**0.034**
Role difficulties	88 (0 to 100)	100 (50 to 100)	**<** **0.001**	88 (38 to 100)	0.389
Dependency	100 (17 to 100)	100 (92 to 100)	**0.028**	100 (33 to 100)	0.052
Driving	75 (36 to 100)	86 (50 to 100)	**0.001**	88 (0 to 100)	**0.048**
Color vision	100 (100 to 100)	100 (100 to 100)	NA	100 (75 to 100)	NA
Peripheral vision	100 (25 to 100)	100 (100 to 100)	**0.005**	100 (50 to 100)	0.778
NEI VFQ-25 composite score	87 (53 to 98)	96 (89 to 100)	**<** **0.001**	89 (59 to 100)	0.156

*SD* standard deviation, *NA* not applicable, *NEI VFQ-25* National Eye Institute Visual Function Questionnaire-25 items, *Preop* preoperative consultation, *Postop* postoperative consultation.

^†^*p* value between Preop and 6 months Postop; ^‡^*p* value between Preop and 2 years Postop; **in bold**: statistically significant difference (*p* < 0.05).

## Discussion

Removal of uiERM results in short-term and long-term improvements in monocular and binocular visual function and VR-QoL. Binocular distance BCVA, vertical metamorphopsia, vertical aniseikonia and stereoscopic vision share the same evolution dynamic (significant improvement at 2 years but not at 6 months postoperatively), thus confirming a slow and progressive clinical recovery of these parameters after surgery.

### Clinical and paraclinical outcomes

It is well described in the literature that metamorphopsias generated by ERMs are greater in the horizontal direction compared to the vertical direction^8,15,16^. According to Chung et al. 2015, metamorphopsia and retinally-induced aniseikonia share the same pathophysiology^17^. Retinal displacement caused by ERM is limited in the horizontal direction due to the presence of the optic nerve head. When the retina contracts vertically, the photoreceptor layer is compressed vertically; the non-uniformity of the distribution of photoreceptors causes horizontal metamorphopsia while the difference in density of the photoreceptors causes vertical aniseikonia. Endorsing this hypothesis, Ichikawa et al. found a correlation between the tangential displacement of the retina and the metamorphopsia score, as well as between the metamorphopsia score and that of aniseikonia^11,15^. In the current study, horizontal metamorphopsia significantly decreased at 6 months postoperatively whereas vertical metamorphopsia decreased at 2 years but not at 6 months postoperatively. There are inconsistent findings in the literature about the evolution of vertical metamorphopsia following surgery^10–12,18^. None of these studies had a follow-up period above 1 year but our results suggest that follow-up duration influences vertical metamorphopsia which improves progressively over years.

Similarly to our findings, it is well known that ERMs are more often responsible for macropsia than micropsia probably because ERM causes centripetal macular contraction which leads to an increase in the density of photoreceptors and the number of photoreceptors stimulated, as well as the size of the perceived image^6,8,11,13,19,20^. In contrast, central serous chorioretinopathy, macular hole, retinal detachment or macular edema are most often responsible for micropsia owing to retinal stretching rather than macular contraction^21,22^. Yet, there are discrepancies between studies concerning the evolution of aniseikonia following ERM removal^8,11–14^. It is possible that horizontal and vertical aniseikonia do not depend on the same underlying anatomical conditions and have different evolutions after surgery. Aniseikonia variation after surgery is still debated and could be related to two factors: follow-up duration after surgery and symptom duration before surgery. Takabatake et al. 2018, reported vertical aniseikonia improvement 12 months after uiERM removal whereas it did not change 6 months after surgery, suggesting that vertical aniseikonia improvement is a slow process^10^. Some report vertical aniseikonia improvement by 6 months^12,23^ whereas other do not^8,12^. Symptom duration may also account for these differences. Only Han et al. 2016, provided symptom duration in patients with uiERM with a 6 months follow-up after surgery and found that greater improvement of aniseikonia after ERM peeling was achieved in patients with shorter symptom durations^23^. The mean symptom duration reported by Han et al., 2016, was similar to our study (12.9 and 13.6 months respectively). Other studies did not provide symptom duration which may be an important factor to assess in further investigations. It is possible that retinal structural alterations secondary to prolonged macular contraction could occur, leading to non-reversible damage of the photoreceptors. These alterations have been reported in other conditions such as cystoid macular edema secondary to diabetes or retinal vein occlusion^24,25^. An important difference between this study and others evaluating aniseikonia in patients with uiERM is the difference in mean age of the sample studied (mean 72 years), which was greater to other studies (mean age ranging from 64 to 68 years)^8,10–12,14,23^. The current findings suggest that even in older patients, aniseikonia and stereopsis improved. Interestingly, vertical aniseikonia quantitatively decreased following surgery but more patients reported macropsia at the end of the 2-year follow-up. Patients who developed postoperative macropsia, concerned in majority those presenting preoperative micropsia (9 out of 11 with preoperative micropsia). It could be possible that the traction of the epiretinal membrane during peeling could modify the architecture of the retina resulting in an increase of the density of photoreceptors particularly in patients with micropsia. This assumption has to be further confirmed by further investigations.

In our study, stereoscopic vision improved significantly at 2 years postoperatively. We believe that improvement in stereopsis could thus be related to the progressive improvement in clinical parameters (i.e. monocular and binocular distance BCVA, metamorphopsia, aniseikonia). Earlier improvement, at 3 and 6 months after ERM surgery have been reported^5,14^. Okamoto et al. 2015, reported that impaired stereoscopic vision in patients with ERM prior to surgery correlated positively with the severity of metamorphopsia and aniseikonia^1^. In addition to that, preoperative severe aniseikonia^1^ and higher symptom duration^5^ before ERM removal were associated with worse postoperative stereoscopic outcomes.

### Vision-related quality of life outcomes

To the best of our knowledge, this is the first study which evaluates long-term VR-QoL in patients undergoing uiERM surgery. Previous cohorts were composed of small samples (between 20 and 37 eyes) and had a postoperative follow-up of 12 months or less^2,4,6,7,12^. The composite scores (84.8, 95.7 and 86.7 at the different stages of follow-up) were higher in comparison to those of other studies, both pre-operatively and post-operatively (respectively: Okamoto et al. 2009^2^: 66.2 and 77.9 at 3 months; Matsuoka et al. 2012^7^: 73 and 81 at 12 months; Nakashizuka et al. 2019^12^: 75.3 and 82 at 12 months; Ghazi-Nouri et al. 2006^4^: 78.4 and 83.3 at 4 months postoperatively). This can be explained by the psychometric discrepancies of the NEI VFQ-25 questionnaire resulting from its translation into different languages or the differences in initial characteristics according to studies (for example the age of the patients included and the severity of the initial macular syndrome). The intermediate results of our study (6 months postoperatively) that show a significant improvement in the composite score are in accordance with the literature. The improvement of the different subcategories varies between studies, with an improvement in 3 to 10 subcategories at 3, 4 or 12 months postoperatively. The following subcategories were the most frequently improved: “general vision”, distance activities”, “near activities”, “role difficulties”.

VR-QoL scores improvements were more important at 6 months postoperatively compared to 2 years postoperatively. These better intermediate results could be partly explained by patients responding more positively with the idea of “satisfying the surgeon”. Secondly, aging may cause negative intercurrent general health events which could explain the reduction in the "general health" and “eye pain” scores. In addition, this questionnaire was validated for patients with ocular hypertension or glaucoma. Such patients have peripheral visual alterations which are not found in patients with ERM who, in contrast, have central visual impairment. The subcategories "color vision" and "peripheral vision" have no reason to be affected by the presence of an ERM. Although, the NEI VFQ-25 is widely used, more specific questionnaires integrating the functional impact of the macular syndrome, including metamorphopsia, aniseikonia and impaired stereopsis, could be built for patients with ERM. However, to our knowledge, there is no such questionnaire validated and translated in French.

### Limitations

The major limitation of this study is that it was a small cohort although it is the largest among studies evaluating VR-QoL in patients with uiERM. Another weakness of this study is that a number of patients underwent lens surgery after surgical removal of the ERM. This bias would mainly be present for the intermediate results 6 months postoperatively and not for the final results as most surgeries were performed before. In addition, the presence of cataract does not influence the following data: metamorphopsia, aniseikonia, central retinal thickness or ellipsoid line. Including pseudophakic patients or performing cataract surgery before inclusion or systematically performing combined simultaneous surgery could be solutions to this problem but too restrictive in the first case and unethical in the last two.

## Conclusion

Surgical removal of an uiERM results in short-term and long-term improvements in VR-QoL and in both monocular and binocular visual function parameters. The various visual symptoms improve with different dynamics and stereoscopic vision can be restored albeit not to a normal level. Visual impairment caused by uiERM is complex and requires multimodal quantitative assessment of different visual symptoms even though this takes a long time to perform and is difficult to integrate into conventional clinical practice.

## Methods

### Patients

We conducted a prospective, single-center study within the department of ophthalmology at the University Hospital of Tours on patients operated for uiERM between January 2016 and June 2017. The current longitudinal study included 42 patients with uiERM who underwent surgery out of 46 who were assessed preoperatively in a previous transversal study^3^.

### Inclusion and non-inclusion criteria

Patients presenting with an uiERM and visual discomfort and/or a BCVA in the affected eye ≤ 5/10 using the decimal Monoyer scale (≥ 0.3 logarithm of the minimum angle of resolution (logMAR)) were included.

Non-inclusion criteria were as follows: ERM secondary to an inflammatory or vascular ocular pathology or appearing as a result of retinal detachment or retinal tear; co-existing ocular pathology (except cataracts) of the eye affected by ERM or the fellow eye ; history of strabismus or amblyopia, and absence of normal retinal correspondence (Bagolini striated glasses test) ; history of vitreoretinal surgery ; bilateral ERM ; distance BCVA ≤ 4 /10 (≥ 0.4 LogMAR or ≤ 6/15) in the fellow eye ; refractive anisometropia defined by a difference in spherical equivalent > 2 diopters between both eyes ; axial anisometropia defined by a difference in axial length ≥ 1 mm between both eyes.

This study complied with the ethical recommendations of the Declaration of Helsinki and obtained approval from the clinical-research ethics board of the University Hospital of Tours (registered under the reference 2016-A00252-49). Written informed consent was obtained from all patients. Data was anonymized in an Excel file, in accordance with Law No. 78-17 of January 6, 1978 relating to information technology, files and liberties. A declaration was made to the National Commission for Information, Technology and Civil Liberties.

### Data collection

The following data were collected: age at the initial consultation, age at the time of the operation, gender, duration of symptoms, follow-up duration and intercurrent eye events. All patients received a clinical examination a month before surgery and postoperatively with an ophthalmologist and an orthoptist. During these consultations the following examinations were carried out:

- Visual acuity: monocular (affected and fellow eye) and binocular distance BCVA was measured with the Monoyer decimal scale and then converted to LogMAR.
- Metamorphopsia: the severity of metamorphopsia was quantified by the M-CHARTS test, originally described in 1999 by Matsumoto et al*.* The procedure is described in a previous study^3^. From the average of the vertical and horizontal metamorphopsia scores, the average metamorphopsia score was calculated.
- Aniseikonia: aniseikonia was quantified with the New Aniseikonia Test (NAT), developed by Awaya et al*.* in 1982. It is considered a reliable method for measuring retinal aniseikonia in symptomatic patients presenting an ERM. The procedure is described in a previous study^3^. For each patient, a vertical and horizontal aniseikonia score was measured. From the average of the vertical and horizontal aniseikonia scores, the average aniseikonia score was calculated. Aniseikonia of + 1% or greater was considered macropsia and aniseikonia of -1% or less as micropsia.
- Stereopsis was measured with the Netherlands Organization for Applied Scientific Research (TNO, ranging from 15 to above 480 s of arc), carried out at a viewing distance of 40 cm with appropriate optical correction. If the stereoscopic vision of the patient was not measurable, the following logarithmic value above 480 arc-seconds was used (progression of 0.3 log arc-second)^26^. The quality of stereoscopic vision was divided into three groups: none" (> 480), "poor quality" (> 120 and ≤ 480) and “good quality" (≥ 15 and ≤ 120).
- Ophthalmologic examination using a slit lamp to observe the anterior segment and a fundoscopic examination of both eyes.
- Spectral Domain Optical Coherence Tomography (SD-OCT): SD-OCT images were obtained using the 3D OCT-2000 Series or DRI OCT Triton (Topcon) with sections covering 6 × 6 mm of the macular region. The ellipsoid zone integrity was considered normal in the absence of disruptions. The procedure is described in a previous study^3^.
- VR-QoL using the National Eye Institute Visual Function Questionnaire-25 items (NEI VFQ-25)^27^. In this study, we used the French version of the NEI VFQ-25, validated in patients treated for ocular hypertonia and chronic open-angle glaucoma^28^. This questionnaire contains 25 questions (items) regarding 12 sub-categories: 11 relating specifically to vision and one on general health. For each subcategory, a score between 0 (lowest capacity) and 100 (best capacity) is calculated. The composite score corresponds to the mean of the 11 subcategories related to vision. The questionnaire was explained to the patient by an investigator (orthoptist, nurse or doctor) and then filled by the patient in the waiting room.

Patients presenting cataract or posterior capsular opacification in any of both eyes at the postoperative consultation received another consultation (between 1 and 2 months later) following appropriate treatment. The surgeon evaluated lens opacification preoperatively and scheduled combined surgery if lens opacification was considered to be clinically significant.

### Surgical protocol

The same surgeon performed all operations, using the same technique, under loco-regional anesthesia, with a conventional contact lens. First, a central posterior 3-way 25-gauge sutureless pars plana vitrectomy was performed. After intravitreal injection of trypan blue dye, which facilitates the visualization of the ERM, a peeling of the latter was carried out in a radius covering the 5 mm centered by the fovea, using "Eckardt" microforceps. No additional peeling of the internal limiting membrane, air or gas intravitreal tamponade or endolaser was performed. Patients presenting a cataract at the preoperative consultation received combined cataract and ERM surgery during the same intervention. Cataract surgery by phacoemulsification and the placement of an intraocular lens was performed first.

### Statistical analyses

Data analysis was performed using Statistica software (version 13.1, Dell^®^). Quantitative variables were expressed as mean ± standard deviation (SD) or as median (range). Data distribution was considered not normal and so nonparametric tests (Wilcoxon and McNemar) were performed, in order to compare the quantitative and qualitative variables respectively. A *p* value < 0.05 was considered statistically significant.

## Data Availability

The datasets generated during and/or analyzed during the current study are available from the corresponding author on reasonable request.

## References

[1] Okamoto F, Sugiura Y, Okamoto Y, Hiraoka T, Oshika T (2015). Stereopsis and optical coherence tomography findings after epiretinal membrane surgery. Retina Phila Pa.

[2] Okamoto F, Okamoto Y, Hiraoka T, Oshika T (2009). Effect of vitrectomy for epiretinal membrane on visual function and vision-related quality of life. Am. J. Ophthalmol..

[3] Khanna RK (2021). Monocular and binocular visual parameters associated to vision-related quality of life in patients with epiretinal membrane: A prospective cohort. Graefes Arch. Clin. Exp. Ophthalmol. Albrecht Von Graefes Arch. Klin. Exp. Ophthalmol..

[4] Ghazi-Nouri SMS, Tranos PG, Rubin GS, Adams ZC, Charteris DG (2006). Visual function and quality of life following vitrectomy and epiretinal membrane peel surgery. Br. J. Ophthalmol..

[5] Asaria R, Garnham L, Gregor ZJ, Sloper JJ (2008). A prospective study of binocular visual function before and after successful surgery to remove a unilateral epiretinal membrane. Ophthalmology.

[6] Okamoto F, Okamoto Y, Fukuda S, Hiraoka T, Oshika T (2010). Vision-related quality of life and visual function after vitrectomy for various vitreoretinal disorders. Investig. Ophthalmol. Vis. Sci..

[7] Matsuoka Y (2012). Visual function and vision-related quality of life after vitrectomy for epiretinal membranes: A 12-month follow-up study. Investig. Ophthalmol. Vis. Sci..

[8] Okamoto F, Sugiura Y, Okamoto Y, Hiraoka T, Oshika T (2014). Time course of changes in aniseikonia and foveal microstructure after vitrectomy for epiretinal membrane. Ophthalmology.

[9] Tanikawa A, Shimada Y, Horiguchi M (2018). Comparison of visual acuity, metamorphopsia, and aniseikonia in patients with an idiopathic epiretinal membrane. Jpn. J. Ophthalmol..

[10] Takabatake M, Higashide T, Udagawa S, Sugiyama K (2018). Postoperative changes and prognostic factors of visual acuity, metamorphopsia, and aniseikonia after vitrectomy for epiretinal membrane. Retina Phila Pa.

[11] Ichikawa Y, Imamura Y, Ishida M (2018). Associations of aniseikonia with metamorphopsia and retinal displacements after epiretinal membrane surgery. Eye Lond. Engl..

[12] Nakashizuka H (2019). Prospective study of vitrectomy for epiretinal membranes in patients with good best-corrected visual acuity. BMC Ophthalmol..

[13] Moon BG, Yang YS, Chung H, Sohn J (2020). Correlation between macular microstructures and aniseikonia after idiopathic epiretinal membrane removal. Retina Phila Pa.

[14] Okamoto F (2020). Preoperative aniseikonia is a prognostic factor for postoperative stereopsis in patients with unilateral epiretinal membrane. Graefes Arch. Clin. Exp. Ophthalmol. Albrecht Von Graefes Arch. Klin. Exp. Ophthalmol..

[15] Ichikawa Y, Imamura Y, Ishida M (2017). Metamorphopsia and tangential retinal displacement after epiretinal membrane surgery. Retina Phila Pa.

[16] Rutstein RP, Currie DC (2019). Topical review: Retinally induced aniseikonia. Optom. Vis. Sci. Off. Publ. Am. Acad. Optom..

[17] Chung H (2015). Relationship between vertical and horizontal aniseikonia scores and vertical and horizontal OCT images in idiopathic epiretinal membrane. Investig. Ophthalmol. Vis. Sci..

[18] Tachibana T (2015). Differential improvement of vertical and horizontal metamorphopsia scores after epiretinal membrane vitrectomy with ILM peeling. Acta Ophthalmol. (Copenh.).

[19] Sayegh RG (2010). High-resolution optical coherence tomography after surgery for vitreomacular traction: A 2-year follow-up. Ophthalmology.

[20] Rutstein RP (2012). Retinally induced aniseikonia: A case series. Optom. Vis. Sci. Off. Publ. Am. Acad. Optom..

[21] Hisada H, Awaya S (1992). Aniseikonia of central serous chorioretinopathy. Nippon Ganka Gakkai Zasshi.

[22] Frisén L, Frisén M (1979). Micropsia and visual acuity in macular edema. A study of the neuro-retinal basis of visual acuity. Albrecht Von Graefes Arch. Klin. Exp. Ophthalmol. Albrecht Von Graefes Arch. Clin. Exp. Ophthalmol..

[23] Han J, Han S-H, Kim JH, Koh HJ (2016). Restoration of retinally induced aniseikonia in patients with epiretinal membrane after early vitrectomy. Retina Phila Pa.

[24] Lardenoye CW, Probst K, DeLint PJ, Rothova A (2000). Photoreceptor function in eyes with macular edema. Investig. Ophthalmol. Vis. Sci..

[25] Murakami T (2007). Photoreceptor status after resolved macular edema in branch retinal vein occlusion treated with tissue plasminogen activator. Am. J. Ophthalmol..

[26] Adams WE, Leske DA, Hatt SR, Holmes JM (2009). Defining real change in measures of stereoacuity. Ophthalmology.

[27] Mangione CM (1998). Psychometric properties of the National Eye Institute visual function questionnaire (NEI-VFQ). Arch. Ophthalmol..

[28] Nordmann J-P, Viala M, Sullivan K, Arnould B, Berdeaux G (2004). Psychometric Validation of the National Eye Institute Visual Function Questionnaire-25 (NEI VFQ-25) French version: In a population of patients treated for ocular hypertension and glaucoma. Pharmacoeconomics.

